# VidaTalk^™^ patient communication application “opened up” communication between nonvocal ICU patients and their family

**DOI:** 10.1016/j.iccn.2021.103075

**Published:** 2021-06-12

**Authors:** Ji Won Shin, Mary Beth Happ, Judith A. Tate

**Affiliations:** a Betty Irene Moore School of Nursing, University of California – Davis, Betty Irene Moore Hall, 2570 48th St., Sacramento, CA 95817, USA; b College of Nursing, The Ohio State University, Newton Hall #352, 1585 Neil Ave., Columbus, OH 43210, USA

**Keywords:** Augmentative alternative communications, systems, Communication aid, Emotions, Family caregivers, Family-centered nursing, Intensive care, Mechanical ventilation

## Abstract

**Objectives:**

To explore family members’ perceptions of an electronic communication application, VidaTalk^™^, their communication experience, and emotional reactions to communication with mechanically ventilated patients in the intensive care units.

**Research methodology/design:**

Qualitative phase of a mixed-methods study nested within a randomised controlled trial. Family members in the intervention group received the VidaTalk^™^ app as a communication aid during their intensive care stay. Seven family members participated in 18 semi-structured email interviews after discharge between May and December 2018. Interviews were analysed using qualitative content analysis.

**Setting:**

Families were recruited in multiple intensive care units located in one university hospital.

**Main outcome measures:**

Communication experience with the VidaTalk^™^ and emotions while communicating with the patient. Basic qualitative description and constant comparative techniques were used to code and analyse the text using ATLAS_ti (Version 7.5.18).

**Findings:**

The VidaTalk^™^ opened up family-patient communication by allowing clear communication and expanding communication content. Family members felt happy and thankful to communicate with the patient. They also expressed feelings of relief and less frustration and less stress while communicating with the patient. On the other hand, the patient’s ability to express their worries or anxiety sometimes made families feel sad or distressed.

**Conclusion:**

The VidaTalk^™^ was helpful for family-patient communication. The VidaTalk^™^ may help families reduce psychological distress. However, expanded communication with critically ill patients may cause other negative feelings.

## Introduction

Communication is a basic need and the essence of human interaction. Patients in intensive care units (ICU) and their family members experience communication impairment during mechanical ventilation treatment. Placement of an oral endotracheal or tracheostomy tube prevents verbal speech and communication is further limited by physical weakness, restraints, sedation and/or limited cognitive function.

Augmentative and Alternative Communication (AAC) methods and tools have been specifically developed and tested to improve communication for patients who are mechanically ventilated in the ICU. These include low-tech tools such as communication boards and tracheostomy speaking valves and high-tech devices such as specialised computerised communication tools (Ten Hoorn et al., 2016). However, the use of AAC tools for communication between patients and family members in the ICU has not been systematically investigated. For the last few decades, a new generation of communication tools, including electronic communication applications, has evolved. More people are familiar and comfortable with electronic devices such as tablet computers and smartphones. Research needs to keep pace with technology development and changes in user familiarity and preference. Little is known about family-patient communication in the ICU. Prior qualitative studies suggest that family members have insufficient tools and methods for communicating with nonvocal ICU patients (Broyles et al., 2012; Dreyer and Nortvedt, 2008; Dziadzko et al., 2017; Engström and Söderberg, 2004; Karlsson et al., 2010). Despite the lack of support for family-patient communication, families often serve as interpreters for clinician communication interactions with ICU patients who are unable to speak and clinicians tend to rely on families’ interpretation (Engström and Söderberg, 2007; Magnus and Turkington, 2006). However, to our knowledge, there is no published study that prospectively investigated family engagement in communication strategies in the ICU. Moreover, strategies to facilitate communication between family members and mechanically ventilated patients have been largely ignored in the literature (Shin et al., 2020).

Broyles et al. (2012) conducted a retrospective qualitative analysis of field notes, intervention logs and brief interviews of nurses from the Study of Patient-nurse Effectiveness with Assisted Communication Strategies (SPEACS) clinical trial (Happ et al., 2014). The purpose of the study was to describe family members’ involvement with communication tools with ICU patients who were mechanically ventilated and their perceptions of communication with the patients. The results showed that families were generally unprepared for the patient’s inability to communicate and not familiar with AAC tools and strategies. However, family members were interested in learning about AAC strategies and desired the highest level of communication possible with their critically ill family member (Broyles et al., 2012). Families experience negative feelings about the inability to communicate with nonvocal ICU patients, such as emotional distress, feelings of loss and frustration (Broyles et al., 2012; Dreyer and Nortvedt, 2008; Dziadzko et al., 2017; Engström and Söderberg, 2004). These negative feelings may induce psychological symptoms that are common among ICU family caregivers, such as anxiety, depression, or stress-related symptoms. However, there is a lack of knowledge about the linkage between communication difficulty and psychological distress in ICU family caregivers.

We report results from the qualitative analysis of interviews with ICU family members who participated in a mixed-methods pilot feasibility-acceptability study of VidaTalk^™^ (Vidatak, LLC), an evidence-based electronic patient communication application (app). The purpose of the qualitative interviews was to explore family members’ experience of communicating with a patient using the VidaTalk^™^ app and their emotional reactions to communication with the patient during mechanical ventilation.

## Methods

### Research design

**Fig. 1.**
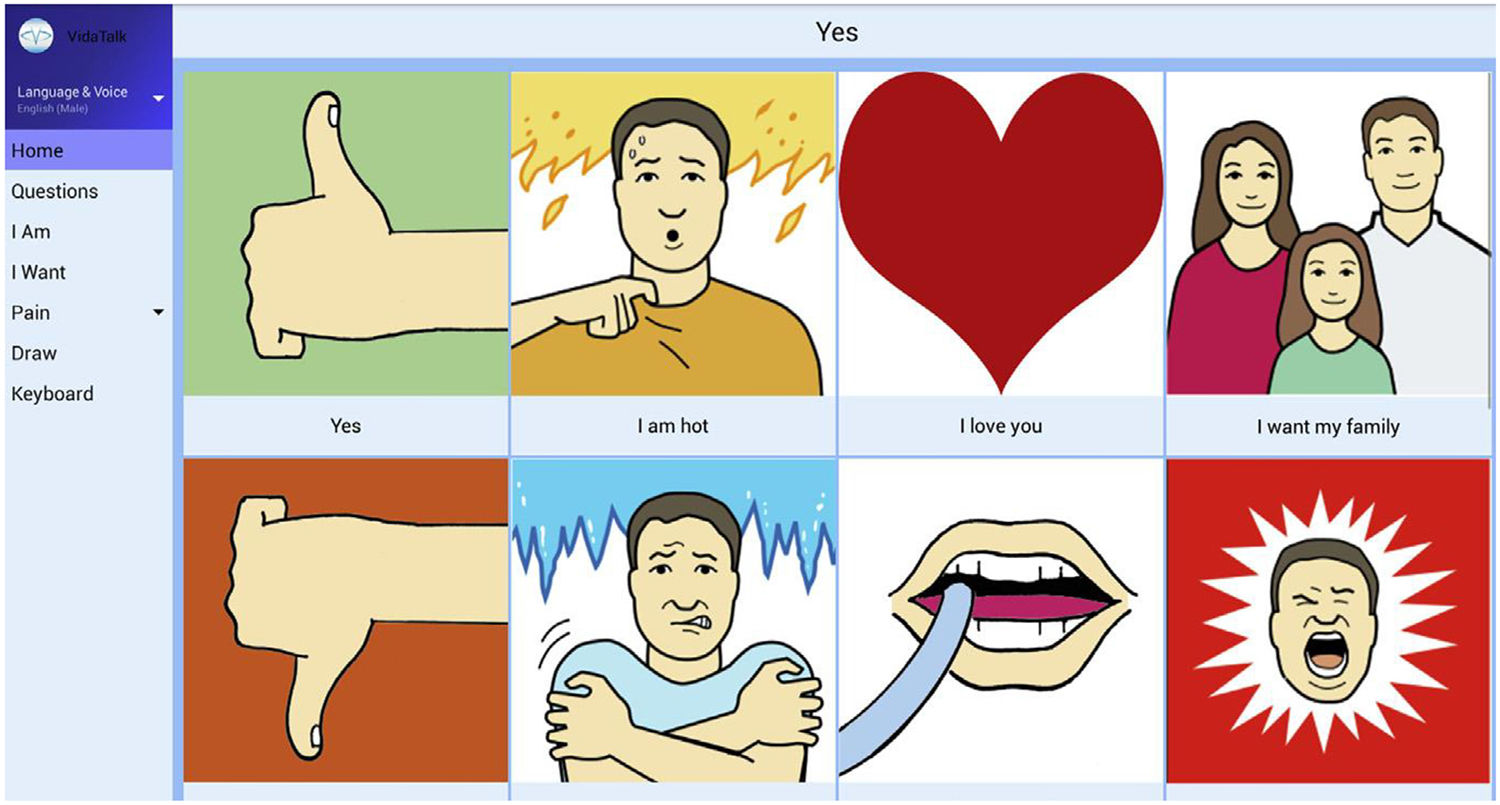
Screen from the VidaTalk^™^ app. Image courtesy of Vidatak LLC. Used with permission.

This study is the qualitative phase of a larger sequential mixed-methods study nested within a randomised controlled trial (RCT). The parent study tested the clinical efficacy of VidaTalk^™^, an electronic communication app for a tablet computer. The VidaTalk^™^ app contains the following features developed for ICU patients: speech-generated messages (common needs, emotions, questions) with picture icons, pain descriptors, finger drawing, and keyboard (Fig. 1). The speech-generating feature allows two-way communication between ICU patients and others.

We recruited family members of patient participants enrolled in the parent RCT study. ICU patients and their family members were randomly assigned to either intervention (VidaTalk^™^) or the attention control group. The intervention group received an android tablet computer with the VidaTalk^™^ app downloaded as a communication aid in addition to the hospital’s standard tablet configuration with the MyChart Bedside (EPIC) application and simple games. Patients and family members in the control group received the android tablet configured with MyChart Bedside application and simple games. Family members in the intervention group only were invited to participate in a series of semi-structured qualitative interviews conducted via e-mail.

### Setting

This study took place at a single 2260-bed university hospital located in the United States. We recruited participants from several types of ICUs: two general medical (36 beds), one medical oncology (24 beds), one general surgical (24 beds), one surgical oncology (12 beds), one general neuro (16 beds), one neuro-oncology (8 beds), one cardiovascular (30 beds), and one coronary care unit (30 beds). The ICU had a nurse-to-patient ratio of 1:2. Standard of care for communication in the ICU at this hospital included writing tools (paper and pen), and occasionally, picture or alphabet communication charts provided at the discretion of the bedside nurse.

### Sample

We recruited eligible family members of ICU patients enrolled in the parent RCT study. Inclusion and exclusion criteria for the family participants are presented in Table 1. Among the 34 family members who participated in the larger study, 18 family participants were assigned to the intervention (VidaTalk^™^) group. Six family members were not eligible for qualitative interviews due to withdrawal or too brief exposure to VidaTalk^™^, and one was lost to follow up after the ICU discharge. Eleven family participants who agreed to be contacted for an interview were invited to participate in the interview after the patient’s ICU discharge or death and before the 1-month quantitative measurement follow-up. The sample size for this qualitative inquiry was not determined a priori or iteratively because recruitment was limited to those enrolled in the larger study.

### Data collection

Qualitative interviews were conducted between May 2018 and December 2018 by the principal investigator (JS) who was then a Ph.D. candidate with prior qualitative research training and experience in conducting qualitative analysis. All procedures and decision-making during data collection were discussed with MBH, a senior scientist with extensive qualitative research experience. Interview participants were asked open-ended questions and follow-up queries about their perception of communication with VidaTalk^™^ using an e-mail interview method. The e-mail interview method was chosen to eliminate travel for a face-to-face interview, to allow participants to reflect on questions, to avoid misunderstanding of phone speech, and to participate at times most convenient to them (Meho, 2006). Moreover, e-mail interviews provided textual data without the need for transcription, which eliminates transcription cost and reliability issues (Hamilton and Bowers, 2006). The e-mail interview involved up to three cycles for each participant. Interview participants received brief information about the interview purpose and instructions on how to use secure email and submit their responses. An email reminder was sent to the participant if we did not receive a response after one-week. We asked participants to respond within a week for each cycle. Two participants chose to end participation after the patient’s death and all others responded to each cycle within a week.

The interview guide (Table 2) elicited the family caregiver’s ideas and opinions about how the use of the VidaTalk^™^ app influenced communication with the mechanically ventilated patient. Questions also addressed families’ emotional reactions to communicating with the patient before and after receiving the VidaTalk^™^ app. The interview questions included individualised probes developed from analysis of the participant’s answers in each previous cycle. Once the participant’s answers were received, the next cycle was sent within three days after the PI reviewed the answers.

In addition to interviews, we collected data from family visitation logs that were completed by family members during the ICU stay. The logs documenting who visited the ICU, the date and time of the ICU visit, and tablet usage.

### Data analysis

Basic qualitative description and constant comparative techniques were used to code and analyse the text (Creswell, 2012) using ATLAS.ti (Version 7.5.18) qualitative data management software. First, the comparison was conducted within each interview by studying and labelling every phrase or sentence of the interview with a code (Boeije, 2002) and constructing tentative code definitions. Codes were added, and existing codes were modified as the analysis continued. The codes and definitions were listed, similar or overlapping codes were collapsed, and codes were arranged into categories. Constant comparison was conducted between interviews by identifying and comparing recurring themes across interviews (Gibbs, 2007). We maintained a low level of interpretation, “data close” approach to coding and analysis consistent with basic qualitative description. Categories and code definitions were revised for accuracy and parsimony after a review of all interview transcripts.

The qualitative data were further analysed by tablet usage categories (high- use and low- use) from the family visitation logs. The qualitative thematic categories were compared within and across tablet usage groups using a stem leaf plot as a form of visual matrix analysis (Miles et al., 2014). Although data saturation was not achieved due to the sample size limitations of the parent study sample, we identified thematic patterns and recurrence of themes within and across participants.

### Trustworthiness

Analyst triangulation was used to assure credibility by engaging multiple analysts (Denzin, 1978; Patton, 1999). Two researchers (JS, MBH) coded three initial transcripts. Both coders compared code definitions and clarification in an iterative process to revise code definitions and code classification continuously. The codes and definitions were reviewed and discussed with faculty experts (MBH, JAT) in qualitative data analysis during regular analysis meetings, which provided credibility and fittingness of findings (Morse and Field, 1995). Involving multiple cycles of e-mail interviews allowed two investigators (JS, MBH) to discuss the responses from each previous cycle and to jointly plan probes for the next interview.

The principal investigator established a relationship with study participants through the recruitment procedure and occasional visits during the participants’ ICU stay. Prolonged engagement with participants during the study period and multiple cycles of qualitative interview strengthened dependability. Additionally, three participants were contacted after the interview completion for clarification of their answers to the interview questions. An audit trail of all analytic decisions and methodologic notes was maintained to strengthen dependability and confirmability (Morse and Field, 1995)

### Ethical approval and human subject protection

The University Institutional Review Board (IRB) (reference number 2016H0006), and the hospital Nursing Department Feasibility Review Committee approved this study. All procedures for recruitment, informed consent, and assessments followed the IRB-approved procedures. The medical centre secure mail system was used for qualitative interviews to minimise the potential risk of loss of confidentiality. E-mail interviews did not contain patient or family participant personal information, other than an e-mail address, and were not linked to any other study data. Text of e-mail interviews was transferred to a word file without identifiable information, and de-identified data were used for data analysis. All data were kept in a password-protected computer on a secured research server.

## Findings

A total of 11 family members were invited to participate in the qualitative interviews; four declined because ‘it was too much’ or ‘I had not enough experience with VidaTalk^™^’ due to a patient’s worsening condition or early extubation. The final interview sample consisted of 18 interviews with seven family members who received the VidaTalk^™^. The demographic characteristics of the participants are described in Table 3.

**Fig. 2.**
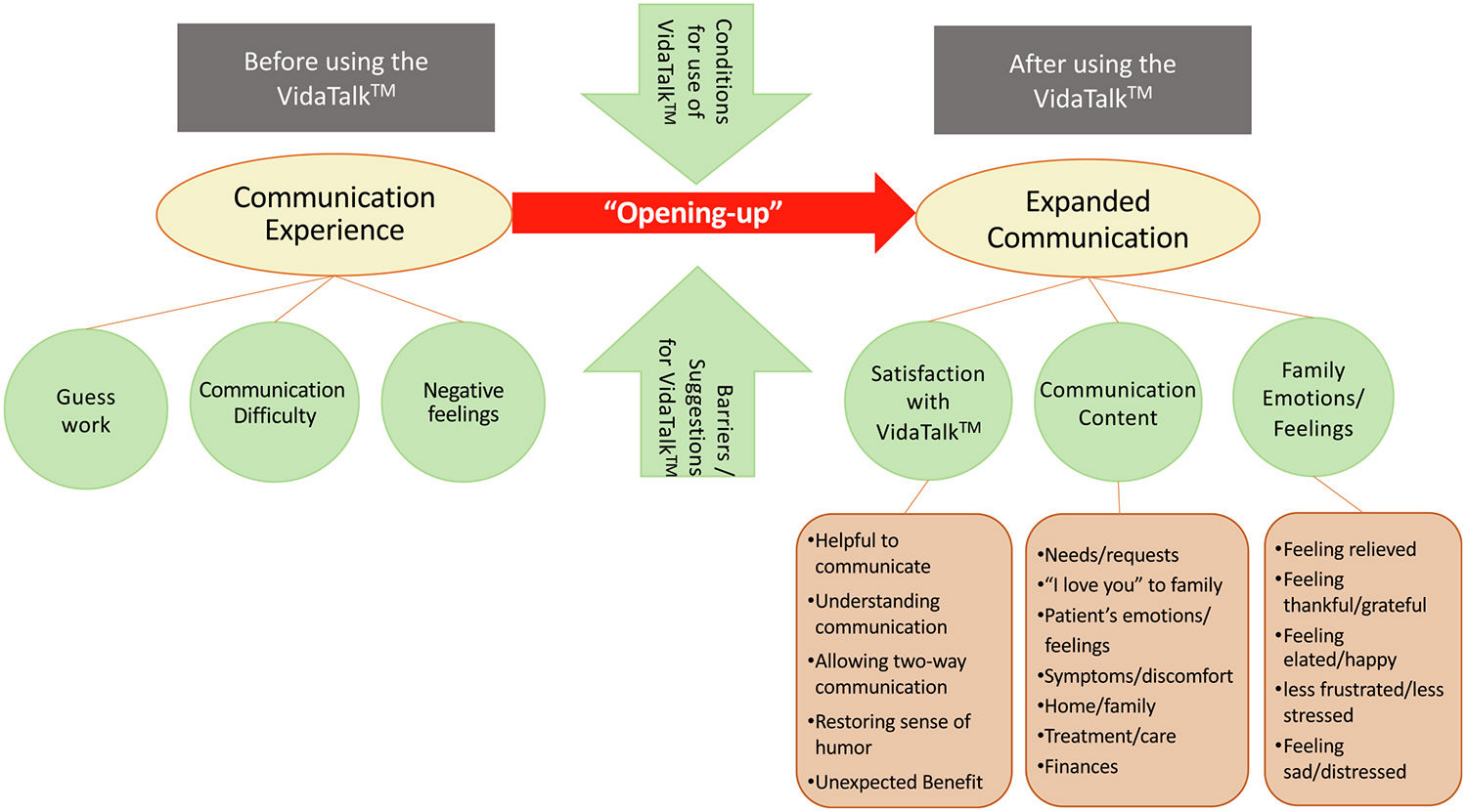
Concept map.

The overarching theme in family members’ experience of patient communication with the VidaTalk^™^ app was an “opening” of communication with the patient and expansion of topics and dialogue (Fig. 2). The opening and expansion were juxtaposed with descriptions of communication experiences before the introduction of the VidaTalk^™^ app.

### Expanded communication

Family members reported that the VidaTalk^™^ app “opened up” the communication with the patient who was mechanically ventilated. VidaTalk^™^ use allowed a broader range of communication between the patient and family member by enabling clearer communication and expanding message content.

“Her ability to use the tablet opened up communication, that was the beginning of a more healthy attitude aiding in her recovery.” ….“After the tablet was in use, communication on all levels was achieved, wants, needs humour, financial, legal, the door was once again opened.”

(Participant 3)

The use of the app enabled an expansion of communication range and functions. The following family comment describes the “opening up” of the patient’s communication ability as greater patient engagement in communication and initiation of communication exchanges. This is contrasted with the patient’s frustration and withdrawal from communication attempts before receiving the VidaTalk^™^ app.

*“Until Tom* (pseudonym) *received the tablet, he didn’t ask as many questions as he then realised he could communicate. He asked about his care more, relayed requests for meds, repositioning in the bed, needing to go the bathroom, who the staff members were that came in groups, need for the fan, a massage. Tom also asked for the music to be turned on or off, asked about the bills, insurance, how our boys and family were doing. He pointed to the icon “I Love You” every day.”*

(Participant 1)

“There was a great difference in communication after receiving the tablet. We all realised that his exact and more detailed thoughts and questions could be discussed. Prior to the tablet use, he was frustrated that he couldn’t be understood and we could visibly see his facial disappointment and exasperation as he waived off any additional attempts to try and relay what he was trying to express.”

(Participant 1)

### Communication experience

Respondents expressed differences in communication before and after using the VidaTalk^™^ app. Most family members described their communication experience with the VidaTalk^™^ app as different and less difficult compared to before receiving it.

*“After having a tracheotomy, my wife and those caring for her found communication quite difficult and frustrating. Attempting to convey the simplest* (sic) *of wants and needs by gesture and communication poster was exasperating. My wife began using a VidaTalk tablet approximately* [date] *with immediate positive results. She enjoys working on her computer and, in little time, was able to navigate the various applications. Within a few days, the tablet was used constantly to communicate with doctors, nurses and visitors.”*

(Participant 3)

#### Communication difficulty and negative feelings before using VidaTalk^™^

Overall, participants described family-patient communication before they were introduced to VidaTalk^™^ as difficult. Family members reported needing to guess what the patient was trying to say by mouthing words, gestures, or writing in the air, but the communication was not always clear. They were not able to understand the patient’s needs or wishes and found reading lips and interpreting gestures to be challenging. The patient was not able to express exactly what she/he wanted to say and the message topics were not easily predicted.

Both families and patients felt frustrated and stressed with the inability to communicate. They described this source of frustration and stress as additional to the situational stress they felt in the ICU.

“I can’t imagine a more frustrating and stress filled situation than witnessing a loved who is in the hospital ICU and unable to communicate clearly.”

(Participant 1)

Families described the patient abandoning or withdrawing from further communication attempts when unable to be understood. The worry and stress that this situation engendered in family members was striking and included concern for the patient’s safety and mental health.

“It was nerve-wracking not to know what my mom was trying to say to us and it also frustrated her.”

(Participant 5)

“We were all worried about his health and the added stress of not understanding his wishes greatly increased his frustration and our worry for his peace of mind, care, and comfort.”

(Participant 1)

#### Communication content

Family members described the content of messages shared with patients using the VidaTalk^™^. The topics most frequently mentioned by family members were the patient’s needs and requests such as repositioning in bed, washing face, or requesting a fan or massage. Patients also reported their symptoms, discomfort, and pain using picture icons or pain descriptors within the VidaTalk^™^ app.

*“I remember her pointing to her legs and bottom on the VidaTalk^™^* [body diagram] *to show us what was bothering her. We were able to let the nurses know where her pain was coming from.”*

(Participant 5)

VidaTalk^™^ enabled more complicated topics such as questions about home/family, finances, and future plans. Patients also asked about things that needed to be addressed with their care, discussed their treatment plan with families, and requested test results. They expressed positive feelings, such as gratitude, as well as negative feelings such as fear, anxiety, or distress about their treatment/care.

*“I remember her pressing the button that said she was scared. She* (patient) *also expressed her feeling of gratitude. She thanked us and told us she loved us on the tablet… Her being able to express feelings of gratitude for those helping her was most rewarding.”*

(Participant 6)

When the patient expressed fear, worries, or feelings of hopelessness, their families provided verbal encouragement and emotional support.

“I was so emotional at that time; Harrison expressed his concerns about his condition and his feeling of hopelessness, and I encouraged him to be strong and everything‘s gonna be OK.”

(Participant 4)

“The conversation with my husband that had the most impact was about his need for a heart transplant. Discussed how strong he was and how the Doctors were so hopeful and impressed with his will and positive mental attitude.”

(Participant 1)

Five of seven family members interviewed stated that their patient said, ‘I love you’ using picture icons or the typing (keyboard) feature within the VidaTalk^™^ app. The ‘I love you’ message from patients to their family was often reported to be the most meaningful conversation with the patient.

“The most meaningful (conversations) were always when we discussed his recuperating, coming home and how much he loved his family. He pointed to the icon “I Love You” every day which means a lot when you can’t hear your spouse’s voice.”

(Participant 1)

### Satisfaction with the VidaTalk^™^

We categorised the family members’ satisfaction with and positive perceptions of the VidaTalk^™^ communication app into four dimensions.

#### Being helpful to communicate

All seven participants commented that the VidaTalk^™^ app was helpful to communicate with the patients during mechanical ventilation treatment in the ICU. The VidaTalk^™^ app allowed patients to express messages clearly, and understandably to family members. It also helped them know whether the patient’s needs were being met.

“The keyboard was nice to be able to type out exactly what she needed or wanted from us.”

(Participant 6)

#### Allowing two-way communication

The VidaTalk^™^ app provided a vehicle for two-way, back and forth communication between the patient, families, and care providers. The communication interactions moved from yes-no questions or simple requests to dialogue.

*“..The tablet allowed him* (the patient) *to type his questions to us* (families) *and we could then answer his questions or relay his wishes to the staff.”*

(Participant 1)

#### Restoring a sense of humour

The VidaTalk^™^ app provided patients with a means to express their personality. Specifically, humour was mentioned as an element of personality and served to lighten the mood.

“With the use of the keyboard, she was also able to joke with those caring for her…Joking was not actually telling jokes per se, but just everyday kidding.”

(Participant 3)

#### Unexpected benefit

One family caregiver described the unexpected rehabilitative benefits of using the tablet. Those benefits included neuromotor coordination in using the VidaTalk^™^ app as well as the psychological benefits of improved attitude, confidence, and well-being.

“Other unexpected benefits of the tablet were improvement of hand-eye coordination and other motor skills such as stretching to reach for the tablet, increasing range of motion.”

(Participant 3)

“Everyone was aware in her overall abilities improving, all centred around being able to talk through the tablet.”

(Participant 3)

### Feelings/emotions while communicating with the patient

All seven participants expressed positive feelings about communicating with their nonvocal family using VidaTalk^™^ (Table 4). On the other hand, several also expressed negative feelings associated with a better understanding of the patient’s feelings. Table 4 shows themes, subthemes, and descriptions and example quotes for each theme.

### Conditions for the VidaTalk^™^ use

Use of VidaTalk^™^ depended on various factors such as patient condition, message complexity, or patient preference. Most patient-family members dyads used a combination of communication methods in addition to the VidaTalk^™^, including unaided methods such as mouthing words/lip-reading, head nodding, gestures, and body language and writing messages using a paper and pen, other communication tools, or finger writing.

When another communication method was the primary method, the VidaTalk^™^ app served a supportive role and was employed under a specific condition, such as a communication breakdown or when conveying a more complicated message. Patients usually attempted a simple communication method, such as mouthing words or hand gestures first, then selected the VidaTalk^™^ app for clarity.

*“We used the tablet* [VidaTalk^™^] *almost daily if we could not read his lips or understand his simple hand gestures. The majority of the time, Tom would try to communicate simple word requests via mouthing words or the use of hand gestures, however, for more wordy communication, he requested the use of the tablet.”*

(Participant 1)

“The patient was able to use the tablet, mostly when she couldn’t get her point across (with other communication methods).”

(Participant 2)

### Barriers and suggestions for future use

Several family members mentioned barriers to using the VidaTalk^™^ app during their ICU stay and suggestions for future use. When the patient’s condition deteriorated, or they were sedated or extremely weak, using the tablet was difficult or impossible.

“It would get frustrating because with him having to stay laying down he wasn’t able to hold the tablet up or there could be days where he didn’t have enough strength to move his arms or fingers in order to tap on the icon that he wanted. It became difficult to hold the device up and still be able to see what icon he wanted to tap.”

(Participant 7)

One family-patient dyad used the VidaTalk^™^ app to express pain/symptoms but preferred using an LCD writing tablet (Boogie Board^®^, Kent Displays Inc) for writing. They found it easier for the patient to write on a Boogie Board^®^ using a special pen.

Most family members recommended the VidaTalk^™^ app for future patients and families. One family member suggested a bedside tablet holder to make using the VidaTalk^™^ app easier for weak patients. The same family member also suggested a training system in the use of communication tools such as VidaTalk^™^ app involving ICU staff and strongly encouraged families and ICU staff to use the communication tool.

### Further comparison of qualitative themes by tablet usage

We examined the number of participants who commented about selected themes by tablet usage group (Table 5). Both high and low tablet users reported satisfaction with the VidaTalk^™^ tool. Both groups reported feelings of relief, less frustration/less stress, and feeling elated/happy while communicating with the tablet. However, participants who were high tablet users experienced more diverse emotional reactions including negative (feeling sad/distressed) feelings. Low users reported more barriers to using the VidaTalk^™^ app to communicate with the patient during mechanical ventilation treatment in the ICU.

## Discussion

We investigated how family members perceived the VidaTalk^™^ communication app while communicating with their nonvocal patient during the ICU stay and their emotional reactions to family-patient communication. This study is the first study that provides a detailed description of the use of an electronic AAC tool between nonvocal patients and family members in the ICU. Although there are several commercial tablet communication app options, peer-reviewed usage and impact data are relatively sparse (Koszalinski et al., 2015, 2016; Nilsen et al., 2018) and the ICU family caregiver perspective is lacking.

The VidaTalk^™^ app “opened up” communication with patients by allowing multiple levels of communication, expanded topic, and perceived better understanding of the patient’s intended messages and needs. Yet, some patient conditions (weakness, sedation) prevented tablet app use. Previous AAC literature also found that patients used more than one communication method and natural methods such as gestures and mouthing words as primary communication methods and for simple yes–no messages (Rodriguez et al., 2016). These findings support the need for a variety of AAC tools (e.g., communication boards, notebooks and markers, electronic apps) and skilled/trained clinicians in a programmatic way so that when patients are unable to use one method, other communication tools are available (Happ et al., 2014, 2015; Trotta et al., 2020).

Family members’ descriptions of communication difficulty and frustration before receiving the VidaTalk^™^ app are consistent with previous studies describing ICU family members’ negative emotions with patient communication difficulty during mechanical ventilation treatment (Alasad and Ahmad, 2005; Broyles et al., 2012; Dreyer and Nortvedt, 2008; Dziadzko et al., 2017; Engström and Söderberg, 2004). Taken together, these studies challenge the common clinical practice of using family members as interpreters of nonvocal communication without providing additional supportive tools and expert guidance from speech language pathologists (Altschuler and Happ, 2019; Engström and Söderberg, 2007; Magnus and Turkington, 2006). Communication between family members and patients may be more stressful than communication with nurses since the patient’s conversation with the family member often took the form of emotional expressions or novel messages (Broyles et al., 2012; Happ et al., 2004a, 2004c).

Davidson (2010) provided examples of caregiving roles and bedside activities for the ICU family member including bringing normalcy into the room (Davidson, 2010; Davidson et al., 2010). Clear communication, as a result of using a communication tool, may promote family caregivers’ bedside activities (Shin et al., 2020). For example, families described knowing, through VidaTalk^™^ communication, that the patient needed to be turned or pain medication. Also, the ability to converse about everyday events outside of the hospital (e.g., home/family) helped the families bring normalcy into the room. Happ et al. (2007) identified the importance of normalising talk between patients and families to distract patients during weaning from mechanical ventilation. Normalising talk referred to talking about every day, non-illness-related events (Happ et al., 2007). VidaTalk^™^ helped the families in our study serve in the caregiver role distracting patients from the stressful ICU environment with normalising talk about home and family.

One of the most frequently reported messages communicated using the VidaTalk^™^ app was the patient’s *“I love you”* message to the family. This finding is consistent with a prior study exploring the use of electronic voice output communication devices with nonvocal ICU patients in which *‘I love you”* was the most frequently observed message (Happ et al., 2004b). Among the seven interview participants, patients of five family members died either during the ICU stay or shortly after the ICU discharge. Although we did not plan to explore end-of-life communication in this study, the families’ communication experience turned out to be end-of-life communication. *“I love you”* is one of the five essential messages in end-of-life communication by dying patients (Keeley, 2007). Considering that these patients were faced with the real possibility of impending death, the *“I love you*” message between the nonvocal ICU patient and the family caregiver was incredibly poignant and meaningful. The VidaTalk^™^ app enabled the patient to express love in a vocal way to the family caregiver.

The meaningfulness of voice output from an electronic communication device and the power of restoration of patient voice has been highlighted in previous research (Happ et al., 2004a). These consistent findings of the importance of the voice between patients and family members over two decades of research indicate that the speech-generating feature of a communication tool may play a crucial role in family-patient communication. However, until the COVID-19 pandemic, tablet computers have not been routinely provisioned as communication devices for patients in most ICUs. The absence of family members in the ICU during the pandemic has also demonstrated the profound importance of patient-family communication during critical illness (Rose et al., 2020). Opportunities to pair communication aids such as the VidaTalk^™^ app with remote patient-family communication should be explored.

Although the families in our study felt relieved, happy, and thankful to communicate with patients using the VidaTalk^™^ app, the patient’s ability to express negative feelings such as worries or anxiety about treatment/care sometimes made families feel sad or distressed. A study of the impact of chronic critical illness on family members found that the patient’s negative emotions such as anxiety, worries, sadness, or distress and pain/discomfort caused family caregiver’s the most perceived distress (Choi et al., 2011). These findings suggest that although a communication strategy may enable and improve family-patient communication, family members may experience psychological distress associated with the negative feelings and worries communicated to them by the patient. The finding that only the high tablet use group experienced negative feelings of sadness or distress is interesting and suggests that implementation of ICU patient communication strategies need to include coaching or debriefing support to family members.

Clinical practice guidelines for support of family-centred care in the ICU (Davidson et al., 2007, 2017) emphasise emotional support for family members by providing more structured interventions to help family members reduce their psychological distress. A communication intervention, such as the provision of the VIdaTalk^™^ tablet app, may enhance family involvement in ICU care, which is an important key to family-centred care (Davidson et al., 2018; Shin et al., 2020).

## Limitations

This study has several limitations to consider. Because of the nested design within the parent RCT, the sample was limited in both size and constituents. Family caregiver’s participation in the qualitative interview after ICU discharge was further limited by post-discharge caregiving burdens and complex challenges. Due to limited recruitment and the small sample size, data saturation was not achieved. However, in this small sample, we were able to explore participants’ experiences from a relatively diverse group in terms of age, sex and relationship to the patient.

We used the email method to conduct semi-structured qualitative interviews. Due to the asynchronous nature of emails, there are several possible limitations such as a lack of non-verbal cues. Traditional face-to-face interview methods require the researcher to listen and think of the next question in real-time during the interview and provide a conversational dialogue that is lacking in email interviews. However, email interviews allow time for reflection in composing follow-up questions and probes. We developed the next set of questions based on the analysis of participant’s answers in each previous cycle.

Transferability of study findings is limited given the small sample recruited from a single university hospital. Also, we relied on a self-selecting sample due to difficulty retaining participants during patient critical illness. Family members who did not participate in the qualitative interview may have different experiences with the VidaTalk^™^ app.

Additionally, we did not conduct qualitative interviews with family members in the attention control (AC) group who received standard care in terms of communication in the ICU which could have provided a qualitative comparison of between-group differences in the communication experience.

## Conclusion

This study contributes to the science of family-centred critical care by adding new knowledge about the process of patient-family communication with and without electronic AAC. VidaTalk^™^ was helpful for family-patient communication, including important end-of-life messages. Family-patient communication through the use of a communication tool such as the VidaTalk^™^ may help reduce family member’s psychological distress. However, families in this study also expressed feelings of sadness and distress with patient’s messages of anxiety and worries. Further exploration of the families’ feelings/emotions and the influence of conversations with the patient on families’ psychological distress is needed.

## Figures and Tables

**Table 1:** Inclusion/Exclusion criteria.

Inclusion Criteria	Exclusion Criteria
Family members of the enrolled patient participants in the parent study as identified by the patient >=18 years old Able to read and speak English Non-professional, un-paid caregiver Identified by patient and/or self as providing the majority of support and visitation Plans to visit > 3 days/week during ICU stay^a^	Severe uncorrected hearing loss^b^ (inability to hear to the audio recording) Self-reported diagnosis of dementia or Alzheimer’s disease Self-reported major psychiatric disorder (bipolar disorder, schizophrenia) or substance abuse requiring inpatient treatment within the last 12 months^c^ Unreliable telephone access^d^ (for follow-up assessment)

a for participants’ exposure to the intervention.

b because the intervention was based on the audio recording feature of the communication tool.

c the main purpose of the study was to assess psychological symptoms of participants.

d reliable telephone access is required for follow-up assessment of participants’ symptoms.

**Table 2:** Semi-structured Qualitative Interview Guide.

Questions	
Cycle 1	We’d like to know about your experience using the tablet computer and VidaTalk^™^ application to communicate with your family member while you were visiting in the ICU. Please tell me about that experience. How often did your family member use the VidaTalk^™^ application to communicate with you or with others while you were visiting? What other methods of communication did you use? Tell me about how you felt when communicating with [patient’s name] after starting to use the VidaTalk^™^?
Cycle 2	4. What messages were communicated using the VidaTalk^™^ application? 5. Tell me about what conversation had the most impact on you? Which conversation was the most meaningful? Which conversation bothered you most?
Cycle 3	6. What suggestions do you have for other family members of patients receiving mechanical ventilation who may use or assist in the use of the VidaTalk^™^ application to communicate?

**Table 3:** Characteristics of participants.

Age	
Median = 61 (interquartile range [IQR], 32) Range 23–71 years	
Sex	
Male	2
Female	5
Race	
Caucasian	5
Asian	1
African American	1
Relationship to patient	
Spouse	4
Sibling	1
Adult Child	2

**Table 4:** Families’ feelings/emotions while communicating with the patient.

Themes	Subthemes	Descriptions	Quotes
Positive feelings	Feeling elated/happy/excited	Several family members described their experience with the VidaTalk^™^ as an ‘exciting’ experience. The families felt ‘happy’ and ‘elated’ that they were able to communicate with their family patients by understanding the patient’s needs and requests clearly.	*“Elated, is the best way to describe my feelings of being able to communicate with my wife.”* (Participant 3) *“It [VidaTalk*^™^*] was very effective in providing the communication my wife needed and we were happy with them.”* (Participant 6) *“Using VidaTalk*^™^ *application is an exciting experience during the difficult time when my husband was admitted in the ICU.”* (Participant 4) *“We were excited to get the tablet and my mom was happy to have a way to communicate with us while having the trach.”* (Participant 5)
	Feeling relieved	Family members felt relieved while communicating using VidaTalk^™^ during their family patient’s MV treatment. The VidaTalk^™^ also relieved the family’s increased worries for the patient’s comfort and feeling of being safe with the inability to understand communication.	*“Once she was able to communicate and know that she had the tools she needed to make us understand her, we felt a sense of relief.”* (Participant 3) *“There was a sense of relief when we found ways for her to communicate with us such as using the VidaTalk*^™^ *app.”* (Participant 5) *“We were relieved to have the ability to communicate clearly*
	Less frustrated/less stressful	Family members reported less frustration with the inability to communicate when using VidaTalk^™^. Some family members believed that they would have felt additional stress without the VidaTalk^™^.	*with my husband -this reduced the additional stress we all felt. I can’t imagine a more frustrating and stress filled situation that witnessing a loved who is in the hospital ICU and unable to communicate clearly. We were all worried about his health and the added stress of not understanding his wishes greatly increased his frustration and our worry for his peace of mind, care and comfort. The tablet eliminated this.”* (Participant 1) *“It was nerve-wracking to not know what my mom was trying to say to us and it also frustrated her. The tablets helped give us a sense of relief to lessen this frustration.”* (Participant 5)
	Feeling thankful/grateful	Family members felt thankful and grateful for participating in this study and having VidaTalk^™^ to communicate with the patient.	*“We thank you for being included in this study and encourage you to share our good fortune with others.*” (Participant 3) *“What I feel after using the VidaTalk*^™^ *to communicate, my husband and I feel thankful that you guys provide this VidaTalk*^™^*. The tablet helps my husband to communicate with me, and during that time, I am thankful and happy that he is able to express his feeling.”* (Participant 4) *“The tablet was greatly appreciated and hope they are offered to all patients who cannot communicate.*” (Participant 1)
Negative feelings	Feeling sad/distressed	Family members reported sadness and/or distress when the patient expressed their anxiety, worries, feeling scared about their health and treatment using VidaTalk^™^. Those conversations bothered family members.	*“Her ability to tell of her anxiety where heartbreaking.”* (Participant 3) *“The conversation bothered me when he said that he’s in pain, he gonna die, he can’t make it.”* (Participant 4) *“The conversation that bothered me the most was his distress about the surgeries.”* (Participant 5)

**Table 5:** Family member’s experience with VidaTalk^™^ by tablet usage subgroups.

Themes		VidaTalk^™^ usage	
		High (n = 4)	Low (n = 3)
Satisfaction with the tool		•••	•••
Feelings/emotions while communicating using VidaTalk^™^	Feeling elated/happy	●●	●●
	Feeling relieved	●	●●
	Less frustrated/less stress	●●	●●
	Feeling thankful/grateful	●●●	
	Feeling sad/distressed	●●●	
Barriers/Suggestions to using VidaTalk^™^		●	●●●

● = number of participants who mentioned the theme during the individual interview.

## Implications for clinical practice

- Family members of nonvocal patients receiving mechanical ventilation treatment experience communication difficulty and negative feelings with the inability to communicate.
- Using VidaTalkTM patient communication application opened up patient communication with families by allowing clear communication and expanding communication content.
- Health professionals need to be aware that increased support for family members is needed because the patients can now better express worries and fears using the VidaTalkTM. Patients’ family members may be more exposed to what the patients are going through, which could be distressing for some family members.
